# Improving mood with psychoanalytic and cognitive therapies (IMPACT): a pragmatic effectiveness superiority trial to investigate whether specialised psychological treatment reduces the risk for relapse in adolescents with moderate to severe unipolar depression: study protocol for a randomised controlled trial

**DOI:** 10.1186/1745-6215-12-175

**Published:** 2011-07-13

**Authors:** Ian M Goodyer, Sonya Tsancheva, Sarah Byford, Bernadka Dubicka, Jonathan Hill, Raphael Kelvin, Shirley Reynolds, Christopher Roberts, Robert Senior, John Suckling, Paul Wilkinson, Mary Target, Peter Fonagy

**Affiliations:** 1 Department of Psychiatry, University of Cambridge and the Cambridge and Peterborough Foundation Trust, Douglas House, 18b Trumpington Road Cambridge, UK; 2 Centre for the Economics of Mental Health Institute of Psychiatry Box P024 De Crespigny Park London, UK; 3 Department of Psychiatry, University of Manchester, Room 4.320 - 4th Floor, University Place, Oxford Road, Manchester M13 9PL, UK; 4 School of Medicine, Health Policy and Practice, Elizabeth Fry Building, University of East Anglia, Norwich, NR4 7TJ, UK; 5 Biostatistics Group Division of Epidemiology and Health Sciences, School of Medicine, University of Manchester, Oxford Road, Manchester M13 9PT, UK; 6 The Tavistock and Portman NHS Foundation Trust, Tavistock Centre, 120 Belsize Lane, London NW3 5BA, UK; 7 Department of Psychiatry, Brain and Mind Sciences Unit, Forvie Site, Addenbrooke's Hospital, Cambridge CB2 2QQ, UK; 8 Department of Clinical, Educational and Health Psychology, University College London, Chandler House, 1-19 Torrington Place, London WC1E 7HB, UK

## Abstract

**Background:**

Up to 70% of adolescents with moderate to severe unipolar major depression respond to psychological treatment plus Fluoxetine (20-50 mg) with symptom reduction and improved social function reported by 24 weeks after beginning treatment. Around 20% of non responders appear treatment resistant and 30% of responders relapse within 2 years. The specific efficacy of different psychological therapies and the moderators and mediators that influence risk for relapse are unclear. The cost-effectiveness and safety of psychological treatments remain poorly evaluated.

**Methods/Design:**

Improving Mood with Psychoanalytic and Cognitive Therapies, the IMPACT Study, will determine whether Cognitive Behavioural Therapy or Short Term Psychoanalytic Therapy is superior in reducing relapse compared with Specialist Clinical Care. The study is a multicentre pragmatic effectiveness superiority randomised clinical trial: Cognitive Behavioural Therapy consists of 20 sessions over 30 weeks, Short Term Psychoanalytic Psychotherapy 30 sessions over 30 weeks and Specialist Clinical Care 12 sessions over 20 weeks. We will recruit 540 patients with 180 randomised to each arm. Patients will be reassessed at 6, 12, 36, 52 and 86 weeks. Methodological aspects of the study are systematic recruitment, explicit inclusion criteria, reliability checks of assessments with control for rater shift, research assessors independent of treatment team and blind to randomization, analysis by intention to treat, data management using remote data entry, measures of quality assurance, advanced statistical analysis, manualised treatment protocols, checks of adherence and competence of therapists and assessment of cost-effectiveness. We will also determine whether time to recovery and/or relapse are moderated by variations in brain structure and function and selected genetic and hormone biomarkers taken at entry.

**Discussion:**

The objective of this clinical trial is to determine whether there are specific effects of specialist psychotherapy that reduce relapse in unipolar major depression in adolescents and thereby costs of treatment to society. We also anticipate being able to utilise psychotherapy experience, neuroimaging, genetic and hormone measures to reveal what techniques and their protocols may work best for which patients.

**Trial Registration:**

Current Controlled Trials ISRCTN83033550

## Background

First depressive episodes tend to arise in vulnerable individuals exposed to current chronic psychosocial adversities and acute adverse life events [1-3]. Later episodes of recurrent disorder are however associated with fewer external stressors suggesting that a history of depressive episodes may itself increase the risk of further illness even in the presence of reduced external adversities [4,5]. Individuals who experience a depressive episode in their adolescent years are at higher risk of recurrence and relapse during their adult life [6]. A history of MD during adolescence is associated with the subsequent emergence of personality disorders and substance misuse in adult life [7,8]. At least 30% of adult affective disorders start in adolescence and thus reducing the duration of an episode and the risk for recurrence would not only reduce short-term morbidity but also help to prevent depressive conditions and suicidal behaviour in later life [9]. Experiencing a depressive episode at any time during adolescence represents a significant health and economic burden on the young person, their family, school and ultimately the gross domestic product of the nation [10,11]. Additionally, depression in adult life is amongst one of the top causes of loss of income to employers in the western world. Therefore, finding ways to improve treatment and decrease the risk of recurrent adolescent depression through adequate treatment of early episodes and reducing the risk for relapse and recurrence would be of a major individual and public health advantage. Currently around 1 in 10 cases referred to CAMHS are identified as having depression so focusing treatment efforts on this severe group may have marked clinical and public health benefits through in adulthood [12].

### Treatments for adolescent major depression

One randomised study in the UK showed that even in adolescent patients who were compliant with full active treatment for 6 months results were moderate; only 20% fully recovered, 30% achieved some level of remission, a further 30% had high number of residual symptoms and 20% did not respond to treatment at all [13]. The possible reasons for such variable responses to apparently adequate treatment well delivered and received are numerous including insufficient treatment dose, higher non compliance than was measured, incorrect treatment choice, failure to select out participants with treatment resistant characteristics and patients who were resistant to current available therapeutics.

Cognitive Behaviour Therapy (CBT) has been widely investigated and shown to be effective in the treatment of mild and moderate depressions in the short term [9,14]. Recent studies reporting treatment of moderate to severe depression episodes have shown that the short term outcomes at 12 weeks for the combination of Fluoxetine and CBT produces some greater clinical improvement than CBT alone [15,16]. Depressed adolescents who were treatment resistant at 12 weeks to an SSRI may show significant clinical improvement with a change to a different SSRI if prescribed with CBT [17]. A recent meta-analysis of the short term (6-12 weeks) outcomes from treatment studies suggested adding CBT to an adequate dose of SSRI provided no statistical improvement for clinical features but some statistical benefit for improving social functioning [18]. As yet no study conducted on depressed adolescents has determined the effectiveness of any psychological treatment on the subsequent risk for relapse and recurrence in the medium term (i.e. 12 -18 months after treatment).

A small study of moderately to severely depressed young people attending clinical services has shown that patients may begin to relapse within 3 months of discharge, even in those fully recovered, and over the next 5-10 years some 50%-70% will relapse with a small group of patients never attaining remission over the third decade of life [19].

There is now a growing evidence base for psychodynamic psychotherapy with adults [20,21] including evidence specifically showing the efficacy of treatment for depressed adults using STPP [22-25]. Relatively few studies have however tested the effectiveness of psychodynamic psychotherapy for children and much of the existing evidence for effectiveness is based on relatively small scale studies [26]. A chart review study [27] of 763 patients included 65 children and adolescents with major depression at the Anna Freud Centre who were treated with long term (average 24 months) psychodynamic therapy showed that by the end of treatment, over 75% demonstrated reliable improvement in functioning and no depressive symptoms. A clear dose-response relationship was also demonstrated with treatment intensity and length of treatment both predicting remission after controlling for level of impairment at referral. A small pragmatic effectiveness trial of psychodynamic psychotherapy versus family therapy for mild to moderately depressed children and adolescents aged 9 through to 15 years showed both treatments were as effective as each other in the short term with a >70% remission rate for either [28] and two other European studies have demonstrated the effectiveness of psychodynamic therapy with depressed children and adolescents [29,30].

Specialist Clinical Care (SCC) refers to the active treatment process that is administered routinely in many but not all UK outpatient child and adolescent mental health services (CAMHS). Specialist Clinical Care is usually delivered through a multidisciplinary team and unlike CBT and STPP is available in the vast majority of all CAMHS across the UK. This treatment approach has recently been manualised and is now being taught to mental health practitioners working in mental health services [31]. To date, no studies have investigated the efficacy or effectiveness of SCC alone or against another psychological treatment. The only UK RCT of adolescent depression conducted in CAMHS clinics has shown however that SCC combined with Fluoxetine is as effective as SCC combined with Fluoxetine and CBT in producing remission at 28 weeks of treatment [32]. This finding supports prior research that SCC could be a psychological treatment of choice with Fluoxetine if an anti-depressant is required as some cases receiving SCC appear to enter remission without medication.

Finally interpersonal psychotherapy (IPT), a conversational treatment with some principles derived from STPP (e.g. therapeutic relationship development, attending to the here and now) and SCC (problem solving in the real world and promoting peer group relationships) has been shown to be efficacious and effective with children and adolescents with mild to moderate depression suggesting that relatively brief, active psychological treatments not focussed on distorted or abnormal cognitive processing treatments are indeed able to alleviate depressive symptoms and improve social functioning at least in the short term [33].

In summary there is now substantial data that 3 active specialist psychological treatments (CBT, STPP, IPT) derived from different theoretical perspectives and requiring therapists to be trained in specific modalities of delivering treatment are efficacious and clinically effective in alleviating depressive symptoms and improving social function in the short term in at least 50% of depressed adolescents. It is likely, but yet to be demonstrated that SCC will have similar properties for the treatment of acute depressive episodes. In contrast there is no evidence that the successful management of acute depression in this age range has longer term benefits through reducing relapse and recurrence risk.

The current randomised controlled trial was designed firstly to determine i) whether psychological treatment delivered to moderately to severe depressed adolescents would reduce risk of relapse and recurrence and if so ii) which was the most likely to reduce the risk of relapse 12 and 18 months after therapy began. This paper describes the design of this trial, how we will implement the protocols and analyse the results.

## Methods/Design

### Overview

The "Improving Mood with Psychoanalytic and Cognitive Therapies" (IMPACT) study is a pragmatic, relapse prevention superiority, randomised controlled trial. The study compares three interventions: Short Term Psychoanalytic Psychotherapy (STPP), Cognitive Behaviour Therapy (CBT) and Specialist Clinical Care (SCC) delivered for the treatment of moderate to severe depression in adolescents attending routine CAMHS clinics in the UK. Patients in any arm may receive Fluoxetine 20-50 mg as part of their treatment for reducing clinical symptoms and improving social function of the presenting episode. The use of an SSRI will adhere to UK NICE guidelines as demonstrated in the ADAPT study [32]. The study will run in three regions: East Anglia, a largely rural area of 3 million people with 4 cities each containing approximately 100,00 people each, North London a densely populated urban sector of the metropolitan London region with around 4 million people and the North West of England covering approximately 4 million people of whom about 1 million are living in rural surroundings with a further 3 million residing in the northern and central sectors of the large metropolitan area of the City of Manchester. Participants will be recruited from around 18 routine CAMHS clinics, 6 clinics within each site. The study design will produce results that will inform which is the most effective treatment in reducing symptoms and improving function in the short term (12 weeks) and the extended medium term (i.e. at 36, 52 and 86 weeks) and what is the most cost-effective treatment. The primary outcome variable is relapse of depression at 86 weeks measured by adolescent self report and by research assessment of current mental state. Treatments will be delivered in the usual clinic settings by staff with training and expertise in one of each of the three modalities. Therapists will not cross-over modalities so that they will only deliver the treatment within which they are qualified.

### Aims

To investigate the clinical effectiveness of psychological treatment in reducing the persistence and/or relapse of major depression in adolescents. In particular, to determine whether the specialist individual psychological treatments of CBT or STPP are more effective at reducing risk for relapse at 86 weeks after treatment had started compared to SCC. We will test a primary superiority hypothesis that STPP and CBT are both independently more effective at reducing relapse risk than SCC and a subsidiary hypothesis that STPP is more effective than CBT.

To investigate the cost-effectiveness of psychological treatment and in particular to determine whether the additional costs of specialised treatment are justified by improvements in effectiveness and/or decreased use of health and social care services by 86 weeks follow up.

### Eligibility Criteria

#### Inclusion criteria

Age 11 through 17 years

Current DSM-IV unipolar MDD diagnoses with moderate to severe impairment.

#### Exclusion criteria

Generalised learning difficulties or a Pervasive Developmental Disorder

#### Pregnancy

Currently taking another medication that may interact with an SSRI and unable to stop this medication.

#### Substance abuse

A primary diagnosis of Bipolar Type I, Schizophrenia and Eating Disorders

No other exclusions will be made in order to ensure that the sample is diverse and representative of the kind of cases that NHS CAMHS take on for treatment.

### Recruitment and baseline procedures

**Figure 1:**
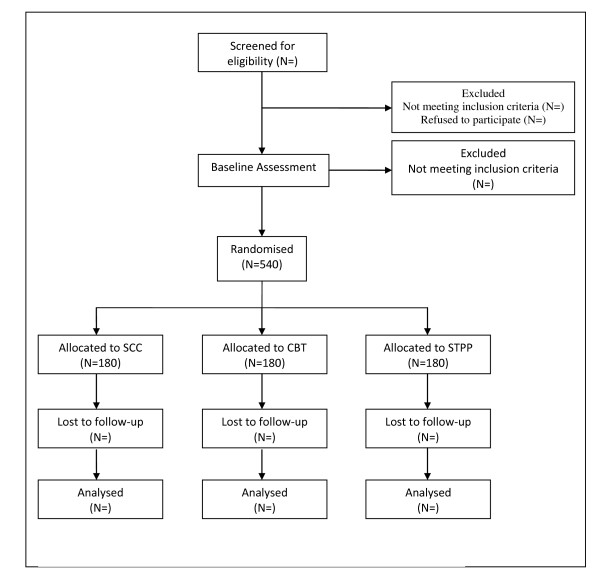
**CONSORT flow diagram of progress though the phases of IMPACT**.

The flow of participants from recruitment through to end of study is shown in figure 1.

The research team will approach local CAMHS within each of the trial sites and will introduce the study to staff allowing them to ask questions and become familiar with the objectives of the trial. Identification and initial screening of potential participants will then be done by clinical staff working within these clinics, following referral as per usual to specialist CAMHS. In many cases the clinicians involved in the initial assessments when the potential participants are referred to a service will be staff who are also delivering treatment as part of the group, based in clinics. At assessment clinicians will be asked to complete a depression screening tool (see appendix) specifically designed to assist referral to IMPACT. Once the potential participants are identified, clinicians will invite the young person and their carer(s) to take part in the trial. If they are interested their details will be passed to the research group.

The participants will then be contacted by a researcher (outcome assessor, OA) who will then schedule an initial meeting during which the participants will be invited to sign a consent form. All research baseline assessments will be administered to the young person and their carer in parallel sessions by two research workers. After this, researchers will confirm whether the participants meet diagnostic and other entry criteria. If they do, participants will then be randomised into one of the treatment arms. Researchers will remain blind to treatment allocation.

### Randomisation procedures and methods to minimise bias

After consent has been obtained and the baseline assessment has been carried out, a trial ID will be assigned. Randomisation will be stratified by age, sex, region and scores on the Mood and Feelings Questionnaire (MFQ) [34]. Randomisation to treatment arms will be done online by the trial co-ordinator, thus ensuring allocation concealment. Information about the treatment allocation will be forwarded to a clinic administrator who will then be responsible for allocating a therapist to the participants. To minimise bias that could arise from knowledge of treatment allocation the following strategies will be employed: a) Outcome assessors will be blind to treatment allocation; b) Outcome assessors and therapist will not directly (if at all) communicate with each other; c) Outcome assessors will be asked to guess which treatment was given so that the effects of possible bias can be examined in the analysis; and d) all outcome assessor interviews will be audiotaped and a random sample re-rated by independent rates. If blindness is broken, all subsequent assessments will be carried out by an alternative assessor.

### Planned Interventions

IMPACT is a pragmatic superiority trial that seeks to evaluate the treatments that could be used in standard NHS practice. Therefore, comprehensive treatment protocols will be used. These are based on usual clinical practice and have been developed for the trial. The rational for using treatment protocols is that: a) protocols aid dissemination of treatment methods into clinical practice; b) they help to standardize the intervention between therapists and across sites; and c) they form the bases for audiotape ratings of adherence to the intervention and thus ensure that the interventions have been given properly; and importantly d) that therapists in the SCC group do not give CBT or STPP interventions. The three treatments will be delivered at different levels of intensity, defined by the total number of sessions over the study period. The number of sessions for each treatment are as follows: SCC 12 individual sessions plus up to 4 family/marital sessions to be delivered over 20 weeks, STPP 30 sessions plus 6 parent sessions to be delivered over 30 weeks and CBT 20 individual sessions plus up to 4 family/marital sessions to be delivered over 30 weeks.

### Specialist clinical Care (SCC)

SCC will consist of a psychosocial management programme with Fluoxetine in severe cases: The procedure will be a treatment course over 20 weeks maximum consisting first of 6 sessions over the first 8 weeks. If remission is not achieved psychosocial treatment will be extended for a further 6 sessions and Fluoxetine added. End of trial treatment will be denoted by week 20 from the first session. The content will involve a conversational approach with the patient and their parents and siblings if required. The treatment will emphasise the importance of action-oriented, goal-focused and interpersonal activities as therapeutic strategies. There will be no focus on changing cognitions and negative cognition-driven behaviours will not be deconstructed. Finally there will be no ongoing analysis with the patient about the putative unconscious origins of their symptoms. Advice will be given on personal activities, social behaviour, and school work and attention will be paid to immediate distressing events such as family difficulties. There will be a continuing focus on psycho-education, i.e. what depression is/co-morbid diagnoses/how common it is/its nature and the typical course of the disorder/how it affects the adolescent and those around them. More detailed attention will be paid to the consequences of any acute undesirable life events focused on the adolescents. Up to 4 family or marital therapy sessions for parents will be given where required within the 20 week period. Liaison with external agencies and personnel e.g. teachers, social care and peer group will be undertaken. Specific advice will be given on mental and physical hygiene. Helping oneself through engaging in pleasurable activities and diminishing solitariness will be strongly enforced.

SCC will not use cognitive or reflective techniques related to analysis of unconscious motives and behaviours nor specific behavioural strategies. Very ill patients may require Fluoxetine before 6 sessions have been delivered. If Fluoxetine is added medical assessment sessions will occur and recorded. The standard protocol developed for the ADAPT study will be used whereby a test dose of 10 mg is given for 48 hours followed by 20 mg. If there is no improvement within 2-4 weeks dose can be adjusted upwards to 60 mg maximum (median dose in the ADAPT study was 30 mg with a range of 20 mg-50 mg). Both adverse events and side effects will be recorded for psychological as well as pharmacological treatments.

### Short Term Psychoanalytic Psychotherapy (STPP)

The form of STPP to be used in the trial, which reflects the approach to psychodynamic work as it is practiced in child mental health services in the UK, has been manualised for the study. This manual draws on the work of other psychodynamic workers [35], including a manual developed by Marie Rhodes and Judith Trowell (unpublished) at the Tavistock Clinic, where the majority of currently practicing STPP therapists in the UK have obtained their training. This treatment manual is specifically aimed at STPP for young people with depression and was recently validated in a multisite trial [28]. The IMPACT manual combines aspects of STPP that focus principally around techniques aimed at helping young people overcome developmental problems using both supportive and expressive strategies by focusing on developmental delays and distortions in children with severe psychological problems. The STPP manual outlines the important role for the interpretation of unconscious conflict, but also makes extensive use of modern attachment theory and the concepts of internal working models. The intervention aims to elaborate and increase the coherence of the young person's maladaptive mental models of attachment relationships and thereby improve their capacity for affect regulation [36]. The comprehensive implementation of STPP also involves parallel work with parents or carers which is also guided by the treatment manual. STPP will be delivered weekly for 30 weeks. Fluoxetine may be added in severe cases as per the protocol used in SCC.

### Cognitive Behaviour Therapy (CBT)

CBT therapy in this trial is based on that developed for adults and has been adapted to include parental involvement and specific techniques for adolescents. CBT is an active, verbal therapy which is based on an individual formulation of the client's current problems and their associated antecedents, precipitating and maintaining factors. This formulation is shared with the client (and their parents).

CBT is typified by an emphasis on 'collaborative empiricism', explicit, tangible and shared goals and clear structured sessions. Typically therapy has a number of phases which include assessment, psycho-education into the cognitive behavioural model of depression (e.g. the links between thoughts, feelings and behaviours), the introduction of monitoring methods (e.g. mood, behavioural and thought monitoring), behavioural activation and activity scheduling, linking thoughts, feelings and behaviours, identifying and challenging negative automatic thoughts, developing and reinforcing adaptive thoughts and relapse prevention strategies. Specific techniques have been developed to support therapy and to maintain engagement and optimism for change. Topics introduced within therapy session are extended and supported outside the session by tasks completed by the client between sessions and reviewed at each subsequent session. Delivery of the programme is flexible; typically it may include 12 sessions delivered weekly, followed by 8 biweekly sessions until the end of the treatment. CBT can be delivered to the adolescent alone or to parents and the young person together and this is determined by the formulation.

### Assessments and outcome measures

To maximise the clinical validity of the outcome evaluations, these assessments will be made across multiple domains using multiple methods and sources.

### IMPACT Assessment Battery

#### Interviews

Major depression and co-morbidity - Schedule for Affective Disorders and Schizophrenia for School Aged Children (6-18 years) (k-SADS-PL) [37]

Monitoring suicidal adverse events across the treatment trial - Columbia Suicide Severity Rating Scale (C-SSRS)[38]

Outcomes of child and adolescent psychiatric disorders - The Health of the Nation Outcome Scale for Children and Adolescents (HoNOSCA)[39]

DSM-IV Borderline personality disorder - Zanarini Rating Scale for Borderline Personality Disorder (ZAN:BPD)[40]

Clinical Global Impressions Scale (CGI) [41]

Service use - Child and Adolescent Service Use Schedule (CA-SUS) [42,43]

#### Self Report

Self-report measure of depressive, anxiety, obsessive-compulsive and anti-social behaviour symptoms as well as global self-esteem - The Young Persons questionnaire (YPQ) containing the Mood and Feelings questionnaire (MFQ), revised manifest anxiety scale (RCMAS), short form of the Leyton Obsessional Inventory (LOI), the Rosenberg self esteem scale (RSES) and an antisocial behaviour checklist consisting of 11 items derived from DSM-IV clinical criteria for conduct disorder behaviour checklist questionnaire [34,44-46]

Responses to low mood - Ruminative Response Scale [47]

Self--harming and risk-taking behaviour - The Risk Taking and Self-Harming Inventory for Adolescents (RTSHIA) [48]

Fundamental emotions - Differential Emotion Scale (DES-IV)[49]

Experiences associated with depression - Depressive Experiences Questionnaire (DEQ) [50]

Cognitive vulnerability to depression - Depressed States Checklist (DSC)[51]

Parenting - Alabama Parenting Questionnaire (APQ)[52]

Life events - Life Events Questionnaire (LEQ)[1]

Personality - NEO-Five Factor Inventory (NEO-FFI) [53]

Health-related quality of life - Euroquol (EQ-5D) [54]

Current friendship satisfaction - Friendships Questionnaire [55]

Symptom intensity - Symptom Checklist (SCL:90)[56]

Working Alliance - Working Alliance Inventory - Short form (WAI-S)[57]

Adolescent Integrative measure (AIM), adaptation of the HCAM - Hampstead Child and Adaptation Measure [58]

Family functioning (FAD)[59]

#### Genetic And Hormone Assays At Entry

At baseline, all participants will be asked to collect saliva samples at waking, 30 minutes after waking and again at 22.00 hrs for two consecutive days, for cortisol assay. There is excellent correlation for cortisol levels between plasma and saliva and also between csf and plasma [60]. Levels will be averaged across the two days to reduce the effects of day-day variation. This procedure and assay protocol will be repeated at the 36 week assessment. One further sample of saliva will be collected at baseline for DNA extraction to test for moderating effects of gene variants. A first hypothesis will be to determine the influence of individual differences in serotonergic genes (including triallelic 5-HTTLPR (rs25531) and 5-HT2A (rs7997012) on treatment response.

We will test whether the 5-HTTLPR s allele, the 5-HT2A G allele, and high cortisol predict i) time remission ii) time to relapse iii) differential response to a specific treatment modality. Naturalistic study has shown that higher morning cortisol in depressed adolescents is associated with longer duration of depressive episode regardless of severity and comorbidity at presentation [61]. Since higher cortisol impairs memory retrieval for prior positive events we predict that there will be greater falls in cortisol in participants that are allocated to cognitive-behavioural therapy and psychodynamic therapy than in those allocated to specialist clinical care. This is because SCC has a clinical focus on moderating negative environments and prescribing action strategies (such as more socialising and peer group meetings) rather than changing internal cognitions. We will test whether change in cortisol by 36 weeks will be associated with reduced risk of relapse by 86 weeks.

#### Structure And Function Of Brain Regions Associated With Emotion And Cognition

There is rapidly emerging evidence for distinctive neural structural abnormalities associated with affective disorders in young people compared to other psychopathologies [62]. There is also evidence for neural functions predicting risk of relapse that involve abnormalities in the visual cortical areas as well as the more classical limbic-frontocortical networks [63]. No studies to date however have incorporated neuroimaging techniques within a large RCT of depressed adolescents. In this trial we will test whether structural and functional differences in depressed patients prior to treatment moderate the risk for relapse. We will test whether poor connectivity between amygdala, anterior cingulate and medial prefrontal cortex during depression recovers following treatment. We predict that those who continue to show poor connectivity at 36 weeks after treatment begins, even if they report less depressive symptoms, will be at greatest risk of relapse. We will test whether functional deficits in emotionally valent face perception, memory, behavioral inhibition and variations in activity in the visual cortex during these tasks predict risk for relapse at 36 weeks independently of structural deficits.

To achieve these aims we will recruit 120 participants (40 from each treatment arm) in the East Anglia Region to a magnetic resonance imaging study. Of these 40 receiving CBT following randomization will have a second scan at 36 weeks. In addition 40 age and sex matched controls with no lifetime history of depression will be scanned once only.

Structural MRI datasets (T1-weighted and dual echo sequences) will be obtained for whole brain, multi-channel segmentation and voxel based morphometry and high resolution grey matter volume estimates in limbic and frontal cortical regions of interest defined a priori by a parcellated template image. Diffusion tensor imaging (DTI) data will be analyzed for whole brain analysis of fractional anisotropy measures of white matter integration and tractography of selected axonal tracts mediating connections between limbic and frontal regions of interest. Twelve diffusion-weighted directed volumes and 5 volumes without diffusion-weighting (b = 0) will be acquired. Acquisition time is ~10 minutes.

Functional MRI (fMRI) datasets will be acquired using Echo-planar images (EPI) depicting BOLD contrast at a sampling time (TR) of 2 seconds during performance of three tasks: a facial emotion processing task designed to activate amygdala and other limbic regions and two tasks involving executive and mnemonic processing of emotionally valent stimuli that will additionally activate prefrontal and medial temporal areas. We will also acquire fMRI data with participants in a no-task or resting state to investigate endogenous dynamics and functional connectivity in fronto-limbic circuits. For all fMRI data acquisitions, but especially for proper control of potential cardiorespiratory confounds in analysis of resting state data^52^, the respiratory cycle and the inter-beat interval (RR) in electrocardiographic data will be simultaneously recorded in the scanner. Total acquisition time for all fMRI datasets is ~40 minutes.

### Follow-up assessment

Follow-up assessments will be conducted at 6, 12, 36, 52 and 86 weeks after the start of treatment. To monitor for any adverse events or side effects of therapy, the researchers will complete the Adverse events/Side effect questionnaire at each follow-up assessment throughout the treatment progress [13]. Please refer to Table 1 below for detailed outline of the planned measures for each follow-up point throughout the trial.

**Table 1:** Assessments administered at research baseline and each follow-up point throughout the trial

Assessment points	Interviews	Self Report Questionnaires	Clinician Administered measures
**Baseline** **(0 weeks)**	*Adolescent: *K-SADS, CGAS, C-SSRS, ZAN:BPD,HoNOSCA *Carer: *K-SADS, CGAS, HoNOSCA, CA-SUS	*Adolescent: *YPQ, EQ-5D, RRS, RTSHIA, DSC, DEQ, DES-IV, APQ, LEQ, FAD & Friendships Questionnaire, *Carer: *YPQ, FAD, APQ, LEQ, SCL:90 & Friendships questionnaire	

**6 weeks**	*Adolescent: *K-SADS, CGAS, C-SSRS, HoNOSCA *Carer: *K-SADS, CGAS, HoNOSCA, CA-SUS	*Adolescent: *YPQ, EQ-5D, RRS, RTSHIA & WAI-S *Carer: *WAI-S & SCL:90	*Adolescent: *WAI-S & CGI

**12 weeks**	*Adolescen:t *K-SADS, CGAS, C-SSRS, HoNOSCA *Carer: *K-SADS, CGAS, HoNOSCA, CA-SUS	*Adolescent*: YPQ, EQ-5D, RTSHIA, DSC, DES-IV, APQ, LEQ, FAD, WAI-S & Friendships Questionnaire *Carer: *FAD, APQ, LEQ, WAI-S, SCL:90 & Friendships questionnaire	*Adolescent: *WAI-S & CGI

**36 weeks**	*Adolescent*: K-SADS, CGAS, C-SSRS, HoNOSCA *Carer: *K-SADS, CGAS, C-SSRS, HoNOSCA, CA-SUS	*Adolescent: *YPQ, EQ-5D, RTSHIA & WAI-S *Carer: *WAI-S & SCL:90	*Adolescent: *WAI-S

**52 weeks**	*Adolescent: *K-SADS, CGAS, C-SSRS, HoNOSCA, ZAN:BPD *Carer: *K-SADS, CGAS, HoNOSCA, CA-SUS	*Adolescent: *YPQ, EQ-5D, RTSHIA, RRS, FAD, DSC, DEQ, DES-IV, APQ & Friendships Questionnaire *Carer: *YPQ, FAD, APQ, LEQ, Friendships questionnaire & SCL:90	

**86 weeks**	*Adolescent: *K-SADS, CGAS, C-SSRS, HoNOSCA *Carer: *K-SADS, CGAS, HoNOSCA, CA-SUS	*Adolescent: *YPQ, EQ-5D, RTSHIA, RRS & DEQ *Carer: *None	

### Planned investigations

IMPACT is a pragmatic superiority trial that compares SCC, CBT and STPP to reduce relapse in adolescents with DSM-IV defined moderate to severe depression. Cases with DSM-IV unipolar major depression will be randomly allocated to SCC, CBT or STPP and outcomes will be assessed at planned follow-up points by outcome assessors unaware of the treatment allocation.

### Sample size and Power

The proposed design of the trial will run in 6 CAMHS in each of the three centres, giving 18 clinics with a minimum of one therapist for each treatment modality in each clinic. Because IMPACT will involve more than two treatments, a number of comparisons can be made between treatment groups. We propose to make two: a) the two individual specialist treatments CBT and STPP will be compared; and b) the individual specialist treatments combined will be compared with SCC. A 2.5% two-tailed significance level has therefore been used in the power calculation. Additionally, power has been calculated according to each of the following hypothesis of comparisons: superiority, equivalence or non-inferiority. It has been assumed that 5 points on the self-report MFQ is the minimum clinically important difference. This is approximately 25% of the change in the MFQ scale from baseline to 28 weeks according to the results from the ADAPT trial [13] and is equivalent to a 1 point improvement on 5 of the 33 items of the scale [13]. The ADAPT trial [15] gave an SD for MFQ of 14.6 across follow-up assessments, so that 5 points corresponds to a standardised effect size of about a third (small to medium) and a non-overlap between treatments of approximately 25% [64]. In the ADAPT trial the correlation between baseline and follow-up at 28 weeks was approximately 0.5 and the intra-cluster correlation for therapist was less than 0.01 [15]. With 18 therapist in each treatment modality and 10 followed-up patients per clinic a superiority analysis comparing CBT and STPP will have a power of over 80% provided that the intra-cluster correlation for therapists does not exceed 0.025. By virtue of the increased sample size comparison of the specialist individual treatments (CBT and STPP) with SCC will have greater power. These power calculations assume a cross-sectional analysis, but statistical analysis will be based on a longitudinal data using a linear mixed model. The use of such model will increase the power of the statistical analysis as data is in effect shared across follow-up time points. ADAPT had 92% follow-up at 28 weeks suggesting a recruited sample size of approximately 540 patients.

### Statistical Analysis Plan

All analysis will be according to intention-to-treat principle. Characteristics of the treatment groups will be described at baseline. Preliminary analysis will investigate the pattern of missing follow-up data. The statistical analysis of the primary outcome measure (MFQ) and the secondary measures will estimate the treatment effect using a linear mixed effects models adjusting for pre-specified prognostic variables (baseline severity, treatment centre, co-morbid behavioural and anxiety disorders, sex and age) and the time point of assessment. The model will include subject level random intercept and gradient effects and also random effect for therapist. Ordered categorical secondary outcome measures such as the CGI scales and suicidality rating scales will be analysed using the proportional odds model [65]. A sub-group analysis by severity will also be conducted using a treatment-severity interaction term.

### Economic evaluation

The objective of the economic evaluation is to evaluate the relative costs and cost-effectiveness of the three treatments. Since STPP and CBT are more resource intensive than best practice SCC, their provision requires additional health service resources that could be used elsewhere. To ensure that such resource allocation is cost-effective, it is necessary to demonstrate that the additional resources spent can be justified, either in terms of saving as a result of reduced demand for other services or in terms of gains in effectiveness.

The economic evaluation will take a societal perspective, including the use of all health, social care, education and criminal justice sector resources plus family costs in the form of travel to trial intervention sessions and productivity losses of the primary carer resulting from their child's illness. Economic information will be collected in interview at baseline and all follow-up points using the Child and Adolescent Service Use Schedule (CA-SUS), developed by the applicants in previous research in child and adolescent mental health populations and adapted for the purpose of the current study [42,43]. Data on the trial interventions, SCC, STPP and CBT will be collected from clinical records.

The cost of the trial interventions will be calculated using a bottom-up costing approach [66] which will involve estimation of indirect time spent on individual cases, including supervision, as well as detailed recording of direct face-to-face contacts. Unit costs will be calculated using data on salaries, employer on-costs (National Insurance and superannuation), conditions of service and appropriate administrative, managerial and capital overheads [67]. Nationally applicable unit costs will be applied to all other resources [42,67]. Productivity losses will be calculated using the human capital approach which involves multiplying days off work due to illness by the individual's salary. The human capital approach has been criticised for its inability to consider labour market responses to time off work due to illness and a tendency to overestimate the true cost of productivity losses [68]. To take this into account, the impact of productivity losses will be explored in sensitivity analysis.

Cost-effectiveness will be assessed at the 86 week follow-up and will be measured in terms of quality adjusted life years using the EQ-5D measure of health related quality of life [54]. Cost-effectiveness will initially be explored through the calculation of incremental cost-effectiveness ratios (ICER) [69]. Repeat re-sampling (bootstrapping) from the costs and effectiveness data will then be employed to generate a distribution of mean costs and effects for the two treatments [70] which can be used to calculate the probability that each of the treatments is the optimal choice subject to a range of possible maximum values (ceiling ratio) that a decision-maker might be willing to pay for a unit improvement in outcome. Cost-effectiveness acceptability curves will be presented to explore uncertainty by plotting these probabilities for a range of possible values of the ceiling ratio [71].

### Ethics

The study protocol was approved Cambridgeshire 2 Research Ethics Committee, Addenbrookes Hosptial Cambridge, UK.

## Discussion

The treatment efficacy and effectiveness for unipolar major depressions in post pubertal adolescents has established that combination psychological and SSRI treatment is the therapy of choice for establishing remission in the short term. Acute treatment appears highly satisfactory of around 70% of cases regardless of clinical characteristics. Currently there are very few clinical markers of poor treatment response to help guide clinicians. Severity and at presentation suggests a longer time to recovery but not treatment non response per se [72]. In contrast non suicidal self injury at presentation is a frequent concomitant of depressive disorders in this age range and seems non responsive to either CBT or SSRIs [73]. Thus for those adolescent cases who present with NSSI there is an increased risk of persistent deleterious behaviour which itself increases the risk for subsequent suicidal thoughts and behaviours making it an indirect risk for depression and perhaps relapse in treatment response cases [73]. The striking perspective for adolescent mental health services planning is the inability to denote those depressed young people who are likely to show persistent depressive illnesses either through non response and maintenance of a chronic course into adult life or through a recurrence risk not evident in the presenting clinical features.

The current IMPACT study was designed not only to be informative in terms of effective and cost effective treatment in the short and extended medium term but uniquely in specifically focusing on reducing relapse risk in the longer term. A second and important addition is that one of the treatment arms will for the first time comprehensively test the effectiveness of a short tem psychoanalytically psychotherapy, STPP. Comparing the outcomes of those who received CBT, STPP and SCC will determine the relative clinical and economic benefits associated SCC and STPP. Furthermore, by collecting regular follow-up data throughout treatment and then at 52 and 86 weeks, we will be able to explore factors that might influence treatment response as well as the long term benefits associated with receiving CBT and STPP. Treatments will vary in length as stated within the respective clinical protocols therefore time to relapse will be calculated from the last treatment session given. Additionally, the IMPACT trial will give us the opportunity to examine the productivity losses and gains of each treatment. This will allow for a set of calculations regarding the treatment costs of each arm and therefore the overall economic effectiveness of providing one or more treatments' to prevent relapse.

IMPACT is also the first study of depression in young people to include measurement of variables that can provide insights into the mechanisms that govern treatment response. Studies of depression in adults have attempted to establish genetic biomarkers of liability for treatment response to SSRIs. A recent metananlyses of the three published studies to date involving 2998 treated depressed adults in total found none of the studies reported results that achieved genome-wide significance, suggesting that larger samples and better outcome measures will be needed [74]. Indeed the UK GENDEP study has recently suggested whilst several genetic signals for association with treatment response are worthy of further investigation individual gene contributions to depression are likely to have only minor effects, and very large pooled analyses will be required to identify them. IMPACT is measuring neural intermediate neural phenotypes as well as genes and individual differences in cortisol levels. We will therefore look for main effects of genes on neural structure and function and cortisol levels and change between entry and 36 weeks. We will also explore putative associations between particular clinical phenotypes including suicidality and NSSI and susceptibility genes targeted by other studies including GENDEP and the TORDIA RCT of treatment resistant depressed adolescents conducted in Pittsburgh, USA [75]. Determining if there is genetic moderation of intermediate phenotypes that are associated with treatment response may be more likely reveal how genetic biomarkers operate than studies looking for direct associations with the clinical features.

## List of abbreviations used throughout the paper

ADAPT: Adolescent Depression, Antidepressants and Psychotherapy Trial: The ADAPT Study; AIM: Adolescent Integrative Measure; APQ:Alabama Parenting Questionnaire; CAMHS: Child and Adolescent Mental Health Services; CA-SUS: Child and Adolescent Service Use Schedule; CBT: Cognitive Behavioural Therapy; CGS: Clinical Global Impressions Scale; C-SSRS: Columbia Suicide Severity Rating Scale; DEQ: Depressive Experiences Questionnaire; DES-IV: Differential Emotion Scale; DSC: Depressive States Checklist; DSM-IV: Diagnostic and statistical manual of mental health disorders (4^th^ed); EQ-5D: Euroquol; FAD: The McMaster Family Assessment Device; HCAM: Hampstead Child and Adaptation Measure; HoNOSCA: The Health of the National Outcome Scale for Children and Adolescents; ICER: Incremental cost-effectiveness ratios; ID: Identification; IMPACT: Improving mood with Psychoanalytic and Cognitive Therapies; IPT: Interpersonal Psychotherapy; K-SADS-PL: Schedule for Affective Disorders and Schizophrenia for School Aged Children (6-18 years) present and lifetime; LEQ: Life Events Questionnaire; LOI: Leyton Obsessional Inventory; MD: Major Depression; MDD: Major Depressive Disorder; MFQ: Moods and Feelings Questionnaire; MRI: Magnetic Resonance Imaging; NEO-FFI: NEO-Five Factor Inventory; NHS: National Health Service; NICE: National Institute for Clinical excellence; OA: Outcome assessor; RCMAS: Revised Manifest Anxiety Scale; RCT: Randomised Controlled Trial; RSES: Rosenberg Self-esteem Scale; RTSHIA: The Risk Taking and Self-Harming Inventory for Adolescents; SCC: Specialist Clinical Care; SCL:90: Symptom Checklist; SD: Standard Deviation; SSRI: Selective Serotonin Reuptake Inhibitors; STPP: Short Term Psychoanalytic Psychotherapy; UK: The United Kingdom; WAI-S: Working Alliance Inventory - Short form; YPQ: Young Persons Questionnaire; ZAN:BPD: Zanerini Rating Scale for Borderline Personality Disorder

## Competing interests

The authors declare that they have no competing interests.

## Authors' contributions

IG and PF designed the trial and the multicenter implementation, SB completed the health economics design and contributed to calculating power for economic variables, JH RK MT BD and RS contributed to drafting the grant application with IG and PF. RK, PW and IG wrote the SCC manual, SR wrote the CBT manual, RS and MT facilitated writing of the STPP manual by a group of expert child psychotherapists, CR wrote the statistical and data analytic strategy and together with IG is responsible for the data collation, IG BD PW and ST devised the adverse events and side effects scale and collated measures with JH MT and PF, ST and IG drafted the paper, JS and IG wrote the neuroimaging design, JS designed the structural and functional neuroimaging protocol, and PW and IG designed and drafted the genetics and hormones strategy and protocol. All authors contributed to revisions of the manuscript prior to submission and approved the submitted draft.  
